# Climate-driven marmot-plague dynamics in Mongolia and China

**DOI:** 10.1038/s41598-023-38966-1

**Published:** 2023-07-24

**Authors:** Lei Xu, Qian Wang, Ruifu Yang, Dalantai Ganbold, Nyamdorj Tsogbadrakh, Kaixing Dong, Min Liu, Doniddemberel Altantogtokh, Qiyong Liu, Sainbileg Undrakhbold, Bazartseren Boldgiv, Wannian Liang, Nils Chr. Stenseth

**Affiliations:** 1 Vanke School of Public Health, Tsinghua University, Beijing, 100084 China; 2 Beijing Institute of Microbiology and Epidemiology, Beijing, 100071 China; 3 National Center for Zoonotic Diseases, Ulaanbaatar, 211137 Mongolia; 4 Department of Epidemiology and Biostatistics, School of Public Health, Peking University, Beijing, 100191 China; 5 State Key Laboratory of Infectious Disease Prevention and Control, National Institute for Communicable Disease Control and Prevention, Chinese Center for Disease Control and Prevention, Changping, Beijing, 102206 China; 6 Professional Biological Society of Mongolia, Ulaanbaatar, 14201 Mongolia; 7 Department of Biology, National University of Mongolia, Ulaanbaatar, 14201 Mongolia; 8 The Centre for Pandemics and One-Health Research, Faculty of Medicine, University of Oslo, Oslo, Norway; 9 Centre for Ecological and Evolutionary Synthesis, Department of Biosciences, Faculty of Mathematics and Natural Sciences, University of Oslo, Oslo, Norway

**Keywords:** Ecology, Ecology, Diseases

## Abstract

The incidence of plague has rebounded in the Americas, Asia, and Africa alongside rapid globalization and climate change. Previous studies have shown local climate to have significant nonlinear effects on plague dynamics among rodent communities. We analyzed an 18-year database of plague, spanning 1998 to 2015, in the foci of Mongolia and China to trace the associations between marmot plague and climate factors. Our results suggested a density-dependent effect of precipitation and a geographic location-dependent effect of temperature on marmot plague. That is, a significantly positive relationship was evident between risk of plague and precipitation only when the marmot density exceeded a certain threshold. The geographical heterogeneity of the temperature effect and the contrasting slopes of influence for the Qinghai-Tibet Plateau (QTP) and other regions in the study (nQTP) were primarily related to diversity of climate and landscape types.

## Introduction

Plague, caused by *Yersinia pestis* infections, is a wildlife disease for which rodents are the main host and fleas serve as the vector^1^. Occasionally, the disease affects humans; indeed, over the course of history it has caused three pandemics, killed more than 200 million people, and profoundly impacted human society and the course of history^2^. Currently, the incidence of plague has rebounded in the Americas, Asia, and Africa in tandem with rapid globalization and climate change, making it a severe public health problem^3–5^. From 2013 to 2018, the World Health Organization (WHO) recorded 2,886 cases and 504 deaths, of which 95% came from sub-Saharan Africa^6^, and another 1,878 cases were identified in Madagascar in 2017^7^. Mongolia and China are two more high-risk countries in which occasional scattered cases of human plague have been regularly reported^6,8–10^; in the period of 2001–2020, China recorded a total of 252 cases of human plague and 44 deaths^11^.

*Y. pestis* is maintained in nature through transmission between hematophagous adult fleas and rodent hosts^12^, including gerbils, rock squirrels, ground squirrels, prairie dogs, wood rats, and marmots. When an infected flea leaves the dead body of its rodent host, it may bite humans and cause their infection, whereby sporadic cases, outbreaks, or even large epidemics can result^1^. The plague's clinical manifestations include bubonic, septicemic, and pneumonic forms^13–16^. Without intensive treatment, the lethality of septicemic and pneumonic plague is almost 100% between one and four days following symptom onset^13,15^; however, the higher probability of pneumonic plague leads it to have a higher death rate^17^.

Many previous studies have shown local climate to have significantly nonlinear effects on plague dynamics among rodent communities^18–20^. Stenseth et al.^21^ evaluated the influence of spring warmth and summer rains on plague prevalence among gerbils in Central Asia. They determined that an increase in spring temperatures of approximately 1 degree centigrade would increase the average number of plague-infected gerbils by approximately 59%. Moreover, several cross-sectional studies have suggested that heavy precipitation has a positive effect and increases plague risk, e.g., the rate of prairie dog plague transmission would increase during years of heavy precipitation in North America^22^. Similarly, increased plague incidence was observed among *Rattus* in general and *R. norvegicus* in particular following a period of heavy seasonal rainfall in Vietnam^23,24^. Regarding marmots, one of the key host animals in Mongolia and China, a few studies have been conducted in the Himalayas. Lu et al.^25^ suggested that in Qinghai Province, marmots prefer warm areas with relatively low altitudes and good vegetation. Gao et al.^26^ determined from satellite data that there is an 80%–100% probability of the Himalayan marmot being present in Gansu Province. However, there is yet a lack of research on marmot plague and climate on a large spatial scale.

China and Mongolia share the risk burden of plague epidemics. These two countries contain distribution area for four species of marmot: *Marmota baibacina* (gray marmot), *M. caudata* (long-tailed marmot)*, M. himalayana* (Himalayan marmot), and* M. sibirica* (Tarbagan marmot)*.* These species are mainly distributed in relatively sparsely populated areas in both countries. It has been shown that plague spread faster in regions with low population density and a high proportion of pasture- or forestland compared with urban areas^27^, which emphasize the importance of attention to marmot plague in China and Mongolia. Among the listed marmot species, *M. sibirica,* which lives in both mountain steppe and steppe habitats in relatively low-elevation grasslands (~ 600 to 1200 m)^28^, is notable for having a wide distribution in Mongolia and in Northeast Inner Mongolia in China^29^. Moreover, *M. sibirica* is the main reservoir of *Y. pestis* in Mongolia and is an important source of human infection^30,31^. Of the 73 plague cases reported in Mongolia since 1998, 59% were associated with close contact with infected marmots and 7% with eating raw marmot organs^32^. Regarding *M. himalayana,* its natural range across the Qinghai-Tibet Plateau (QTP) constitutes the largest natural infection area in China; it covers more than 630,000 km^2^ and includes the Qinghai, Gansu, Tibet, Xinjiang, and Sichuan Provinces. The QTP region is a major plague area in China and is particularly vulnerable to the effects of climate change. Over the past several decades, the QTP region has experienced a noticeable warming trend^33,34^. This has led to unstable ecological environment of local foci, resulting in the emergence of dynamic and complex plague patterns^35^. In recent years, the tourism in the QTP region has grown substantially, which further increases the spread risk of plague^36^.

**Figure 1:**
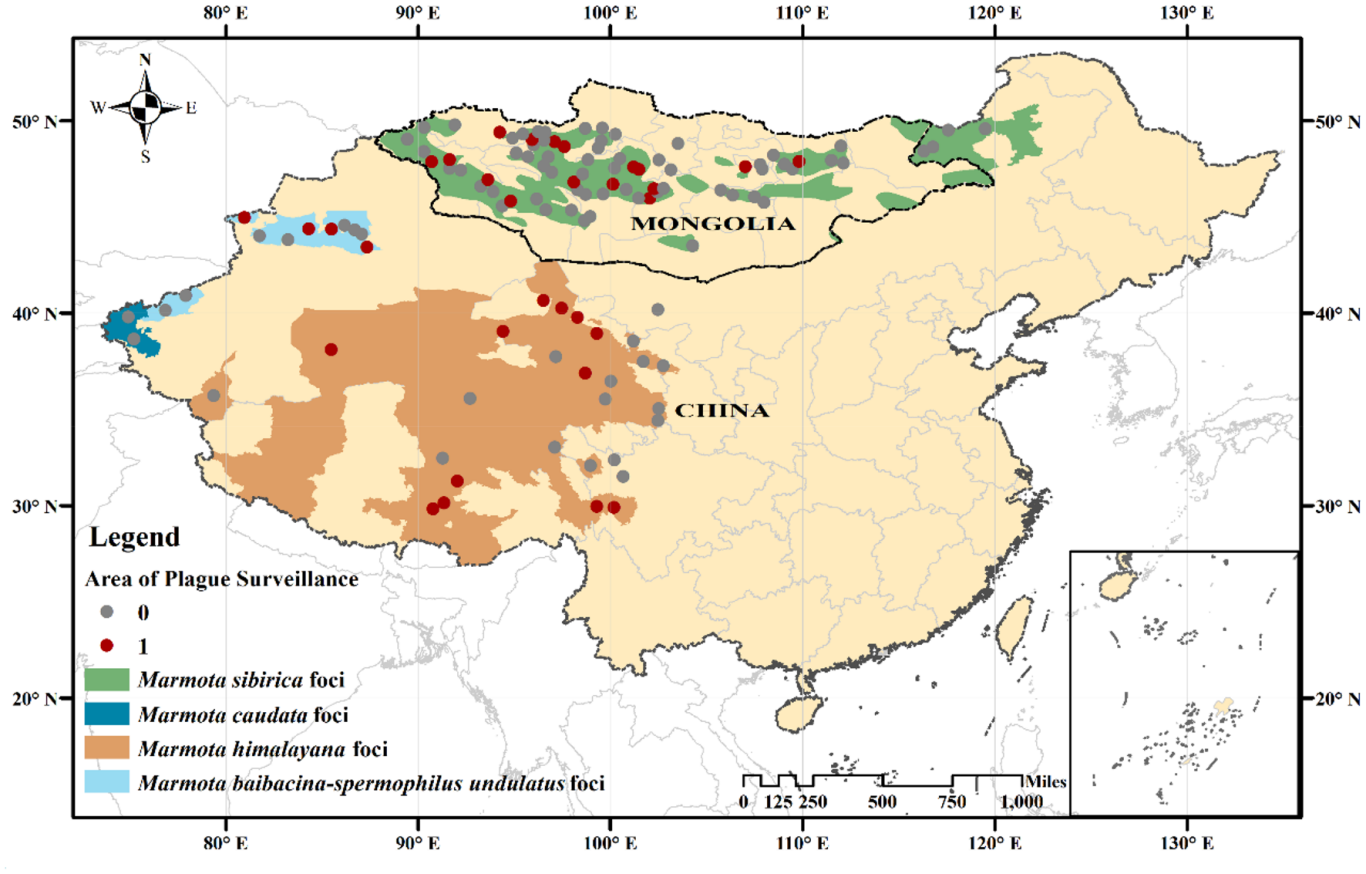
Marmot plague in China and Mongolia during 1998–2015 CE. Circles show areas of plague surveillance (red indicates plague detection at a site during the study period, and gray no such detection). The green, dark blue, orange, and light blue areas represent plague foci for four species of marmots in Mongolia and China^56–58^. *Marmota sibirica* foci (green areas) and *Mamota baibacina* foci (light blue areas) are partially overlapping in Mongolian Altai region. Map was generated with ArcGIS Desktop (ESRI, Inc, Version 16.0, https://desktop.arcgis.com). Boundary shapefiles of China and Mongolia from GADM data (https://gadm.org/data.html).

Here, we analyze an 18-year record database of plague in the marmot regions of Mongolia and China, spanning from 1998 to 2015 (1998–2015 for Mongolia, 2005–2015 for China) (see Fig. 1), to trace the associations between marmot plague occurrence and biological and climatic factors. Our analyses revealed marmot density to influence local precipitation-driven plague dynamics, and also a spatial heterogeneity in the effects of local temperature among marmot foci. We additionally built a threshold generalized additive model (TGAM) that took marmot density as the threshold covariate and included geographical elements, which provided novel insight into the relationship between the plague and marmot populations and climate.

## Results

We used generalized additive models (GAMs) to explore the association between biological and climate factors and marmot plague occurrence. Figure 1 shows the spatial distribution of plague surveillance areas from 1998 to 2015; in total, 526 records were collected, of which 85 reported plague detection. The cases reported were spatially clustered, with *M. sibirica* in Mongolia, *M. baibacina–Spermophilus undulatus* in Xinjiang, China, and *M. himalayana* in Gansu, Sichuan and Tibet, China. Average annual temperature and cumulative annual precipitation were used as climate proxies for historical climate change during the study period, covering 118 plague surveillance areas across China and Mongolia, and we quantified the effects of plague in context of a biological threshold (host density) and geographical restriction to account for spatial correlation (see Materials and methods, Statistical modeling). The uncertainty in our model is depicted as the standard error envelope (see Figs. S2 and S3).

**Figure 2:**
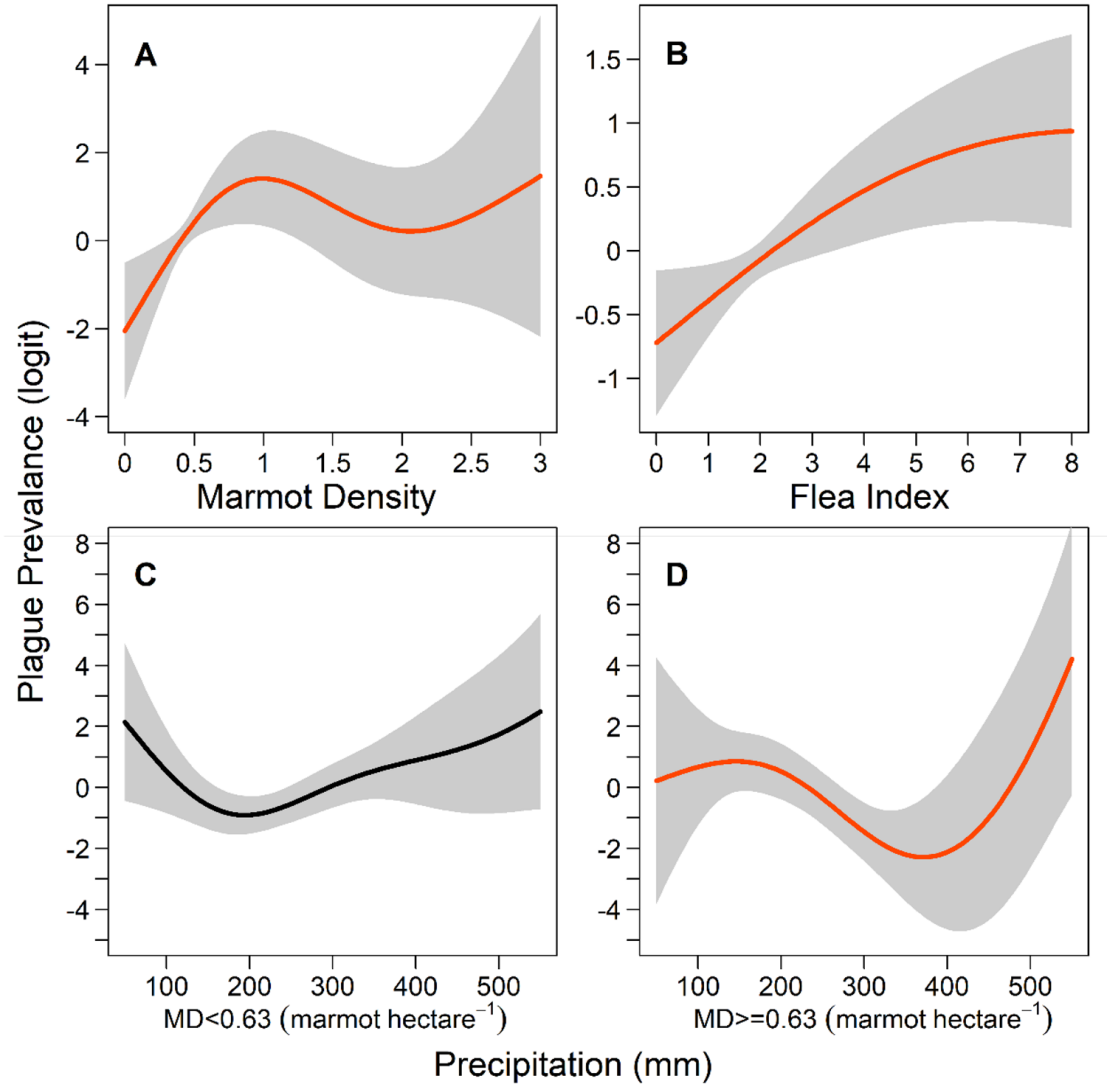
The effect of marmot density on local precipitation-driven plague dynamics. We found partial effects on plague prevalence for marmot population density, flea index, and annual cumulative precipitation. Plots show the relationship between (**A**) marmot density and plague prevalence, obtained from *f*_*1*_ in Formula 1; (**B**) flea index and plague prevalence, obtained from *f*_*2*_ in Formula 1; (**C**) precipitation and plague prevalence when marmot density was < 0.63, obtained from *f*_*4*_ in Formula 1; and (**D**) precipitation and plague prevalence when marmot density was >  = 0.63, obtained from *f*_*5*_ in Formula 1. The red curves in panels A, B, and D indicate *P* < *0.05,* while the curve in Panel C is black because *P* > *0.05*.

The statistical model results indicated a nonlinear effect of marmot density and the flea index on marmot plague occurrence, with an upward trend followed by a flattened trend (*P* < 0.05; see Fig. 2A,B), which indicated the risk of plague occurrence to be significantly associated with both marmot density and the flea index until it reached the inevitable probability of 1. With regard to precipitation, a significant effect on marmot plague was observed in almost all models (see Table S1). We utilized marmot density as a threshold for judging the impact of precipitation on the occurrence of plague. This analysis revealed different interaction curves for precipitation and plague under two different regimes of marmot density (see Fig. 2C,D): when marmot density was below 0.63 (marmot/hectare), the risk of plague did not change significantly with precipitation (*P* > 0.05; see Fig. 2C), but when marmot density was at least 0.63 (marmot/hectare), heavy rainfall increased the risk of plague (*P* < 0.01; see Fig. 2D). This density restriction was also slightly reflected in the effect of temperature on the plague, which we have demonstrated in Formula S1, shown in the *Supplementary Information*.

Regarding the effect of temperature, we found that temperature by itself did not significantly influence the model (see Table S1), which prompted us to explore the geographical heterogeneity of temperature over this expansive research area. In the final model, a synergistic effect of temperature, latitude, and longitude achieved significance (*P* < 0.01). Fig. S2 shows the spatial response pattern of marmot plague occurrence to extremely cold (temperature = − 10 °C), very cold (temperature = − 5 °C), normal (temperature = 0 °C), hot (temperature = 5 °C) and extremely hot (temperature = 10 °C) climatic conditions. Patterns were estimated by a GAM that allowed for spatially variable and nonlinear effects of climate. The results suggest considerable spatial heterogeneities in the effects of climate within the study region.

**Figure 3:**
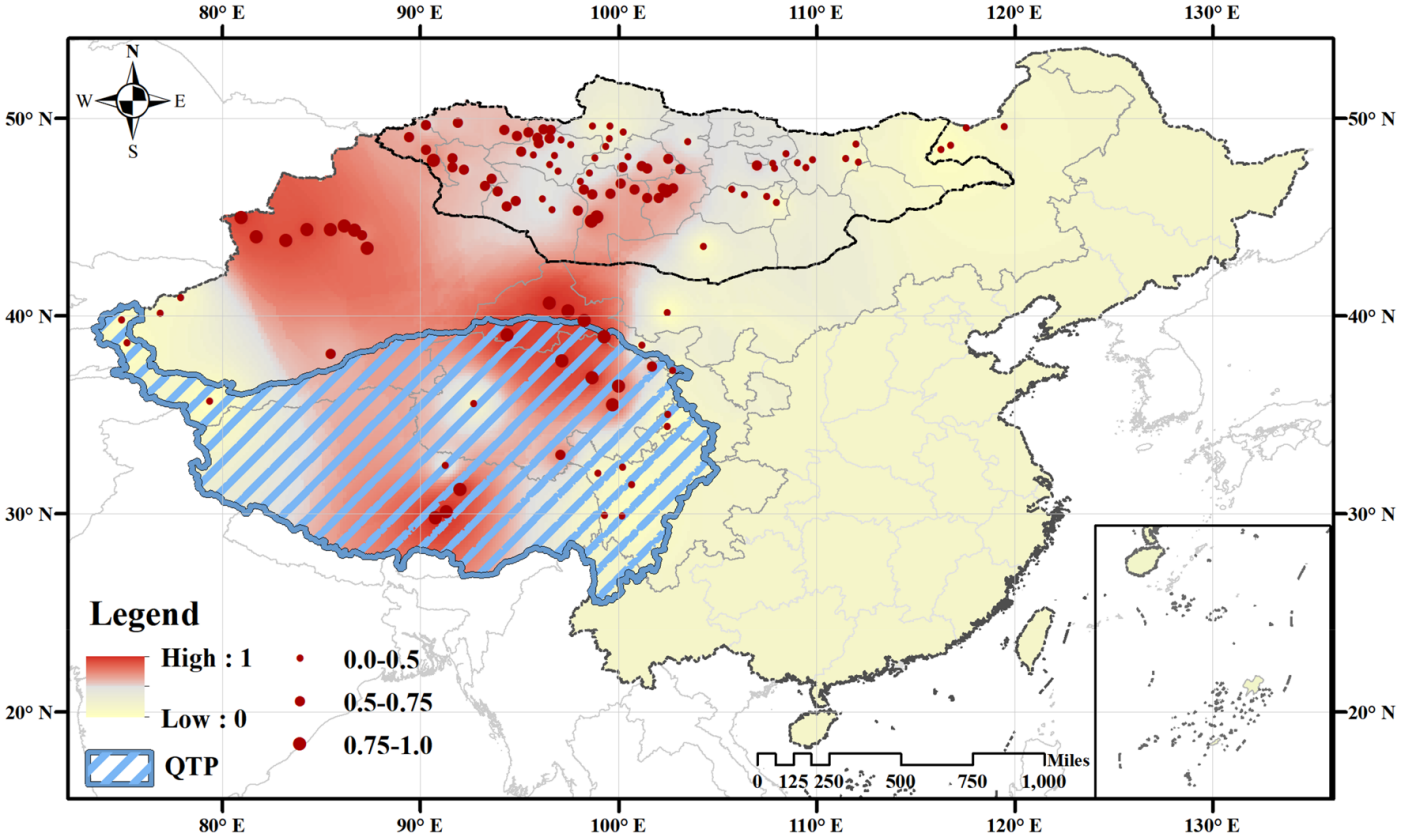
Geographical heterogeneity of the effect of annual average temperature on the risk of prevalence of marmot plague, based on Model (1) *f*_*3*_. We predicted the prevalence of marmot plague cases from the final model. The color gradient represents predicted log-transformed plague prevalence, ranging from 0 (yellow) to 1 (red). Points show the locations of plague surveillance, and the point size indicates the occurrence risk of plague. Blue shading indicates the QTP region, a.k.a., the Third Pole. Map was generated with ArcGIS Desktop (ESRI, Inc, Version 16.0, https://desktop.arcgis.com). Boundary shapefiles of China and Mongolia from GADM data (https://gadm.org/data.html).

Figure 3 reveals the geographical heterogeneity of the effect of average annual temperature on the risk of marmot plague prevalence. Because the predicted risk of plague occurrence ranges between 0 and 1, we were able to depict it as a color gradient provided other factors were fixed and only the effects of temperature were processed in the model. The detailed calculation process can be found in the *Materials and Methods* and *Supplementary Information*. As illustrated by the red areas in Fig. 3, the regions with high risk of marmot plague are located in western China and Mongolia. The standard error of this geographical heterogeneity of temperature is provided in Fig. S2F. This kind of geographical heterogeneity was not found for the impact of precipitation on marmot plague, as provided by Formula S2 in the *Supplementary Information*.

**Figure 4:**
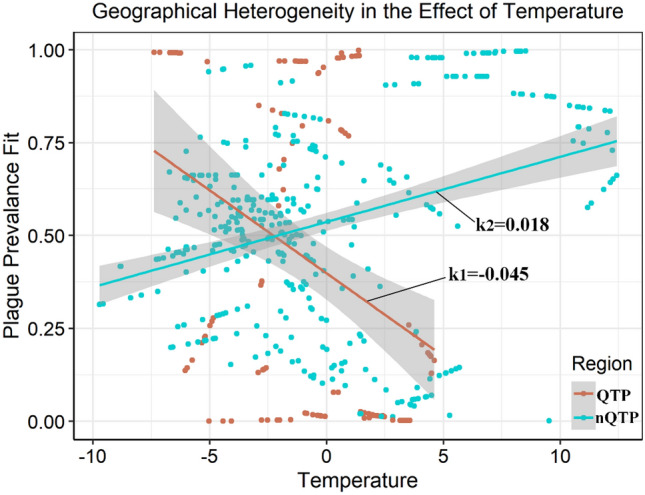
The different effects of annual average temperature on the occurrence of marmot plague in the QTP and other regions plotted in Fig. 3.

Considering the particularity of the topography and climate type of the QTP, we considered the predicted plague prevalence by temperature separately in the QTP and the non-Tibetan Plateau region (nQTP), assigning locations to either area based on latitude and longitude. After processing the partial effect as described in the Methods section, we observed an implication that as temperature rises, the risk of plague occurrence in other areas increases while the risk in plateau areas decreases (Fig. 4). Moreover, the regression parameters illustrate that if temperature increases additively by 1 °C, the risk of plague occurrence will on average increase 4.5% in the nQTP, whereas decrease 1.8% in the QTP of the study period (Fig. 4). As mentioned above, temperature by itself did not significantly influence the model (e.g., Model 5 in Table S1). However, in the counterpart geographically-piecewise model, temperature significantly affected both the nQTP and the QTP (Model 7 in Table S1), which illustrates the strong geographical heterogeneity of temperature effect.

## Discussion

Our results suggest a complex relationship between plague epizootics and biological factors. As marmots and fleas are the primary host and vector respectively in plague spread dynamics, and there are fundamental ecological (ethological) factors of the environment that underlie the formation of a plague epizootic system^37^, it is reasonable to conceive that increases in marmot population density and the flea index (see Materials and methods for definition) would increase the risk of plague occurrence. With marmots being burrowing rodents that live in groups, an increase in marmot density means that the number living in each burrow system increases or that the number of active burrow systems increases. In either case, the probability of the plague spreading within a group or between two burrow systems will increase accordingly. Meanwhile, the flea index, being a measure of risk for epizootic spread^38,39^, positively influences the spreading of plague.

Our final model involves the explicit density of an animal reservoir and showed the interaction effect of climatic covariates and marmot density on the occurrence of plague. Specifically, we observed a positive relationship between risk of plague and high levels of precipitation; however, this effect was significant only when marmot density exceeded a threshold level. Increased precipitation characteristically benefits rodents, especially those living in typically dry habitats; for example, "good years" with high levels of precipitation improve their forage and water balance, which synergistically increases the risk of plague. This is supported by a study that noted the negative effects of the plague in wet years might overwhelm the otherwise beneficial effects of increased moisture^40^. However, other research has also demonstrated that excessive rainfall results in a tremendous decline of rodent population^41^, with high-intensity precipitation causing burrows to flood and many rodents to die^42,43^. Considering these reports, our findings suggest the following “model”: in general, precipitation and plague incidence show a positive correlation, which is consistent with the “plentiful rainfall enhances plague prevalence” theory^21^. This relationship only achieves significance when the number of hosts is sufficiently high (Fig. 2D), which indicates that after a certain number have died (i.e., when the number of marmots is below a threshold), even if heavy rainfall has had a catalytic effect on the plague, it will be difficult for an epidemic to occur because there will not be sufficient host population density. Thus, our study proposes a new threshold theory: a threshold for the biological impact of precipitation on the plague, distinct from the invasion threshold^44–46^ posited by many scholars, in which for the plague to spread, the great gerbil population density and body flea index must be sufficiently high^47,48^.

With regard to the temperature effect, the wide geographical heterogeneity of temperature and the opposite slope of influence observed in the QTP compared to other regions both primarily relate to the diversity of climate types in our selected research area. Studies have validated that *Y. pestis* exhibits prolonged growth at temperatures between 35 and 37 °C, but grows very fast at 28 °C and dies very rapidly if exposed to temperatures exceeding 40 °C or to intensive desiccation^49–51^. Therefore, risk of plague on the QTP will decrease with increasing temperature: the altitude of the QTP dictates a strong correlation between temperature and solar radiation, which inhibits the presence of *Y. pestis*. However, in nQTP regions, increasing temperatures are expected to positively affect plague risk. After removing the effect of strong UV rays, the mechanism is consistent with the traditional idea that increasing temperature may increase the development, incubation, and replication of the *Y. pestis* in the environment^52,53^.

Earth is facing accelerated climate change and globalization, which may pose challenges for plague prevention and control^54^. Our climate is becoming warmer, which may accelerate the spread of plague (via flea transmission efficiency) in nQTP regions (e.g., Mongolia) but slow the spread in plateau regions. With precipitation also becoming more sporadic, we must not be passive about monitoring and recording marmot density, since increased precipitation may increase risk of plague transmission (by benefiting its hosts) in regions where the marmot population is stable. In the case of limited surveillance resources, how to allocate them more reasonably to achieve effective prevention and control becomes particularly important. Firstly, long-term and ongoing monitoring of marmot densities is necessary to ensure we aware whether they are approaching or reaching thresholds. Secondly, different strategies should be adopted to predict and deal with plague risks in plateau and non-plateau areas. Thirdly, once the marmot density exceeds a certain threshold, local governments should proactively intervene in it to reduce the risk of plague outbreaks.

Our study validates the threshold theory in plague transmission not only in the great gerbil (*Rhombomys opimus*) populations in Kazakhstan^44–46,48^ but also in marmot populations in China and Mongolia. This throws new light on how hosts density of other host species in other locations affect plague dynamics. However, given the differences between climate, host species and spatial heterogeneity across locations, future studies should be considered on a case-by-case basis. Altogether, this study provides a different perspective on the heterogeneous effect of climate-driven marmot plague dynamics across Mongolia and China, enhancing our capacity to target potential risk areas, develop disease control strategies, and allocate medical supplies.

## Materials and methods

### Data

#### Marmot plague data

The marmot plague data in Mongolia from 1998 to 2015 were obtained from the National Center for Zoonotic Diseases of Mongolia. The plague data in China from 2005 to 2015 were obtained from the China National Notifiable Disease Surveillance System^20^. Plague surveillance was performed in each country twice a year, in May and July when marmots emerge from hibernation, and the average value of the two measurements was used as the annual result. Host density, flea index, tested rate of seropositivity, and bacterial isolation were determined by the standard protocol described in the National Scheme of Plague Surveillance released by the National Health and Family Planning Commission of the People's Republic of China. For plague occurrence data, marmots were sampled using snap traps. As the results of antibody tests may have a time lag (e.g., rodents infected with plague one year may also test positive for antibodies the next year), the occurrence of marmot plague was determined based on the results of bacteriological tests in order to preclude any adverse effect lag may have on model accuracy; such tests included attempts to isolate *Y. pestis* from blood, spleen, liver, or flea homogenates. Marmot tissue was tested for the plague bacterium using etiological methods from the WHO. The results were processed into a binomial categorization of marmot plague presence (1 = detected in that year, 0 = not detected), and the spatial distributions are visualized in Fig. 1.

#### Host density

The route method was used to determine marmot density in marmot foci. It includes three approaches: walking, horse riding, and car driving. The shape of the route could be a straight line or a curve. According to the National Scheme of Plague Surveillance rules, the width of the field of view is generally 50 m on each side for every method. The route length is 3 km walking or 5 km riding in one hour; when driving, the route length is based on mileage table calculations. Finally, the number of marmots in a hundred square meters was reported as the marmot density. We removed outliers that were outside three standard deviations of the average marmot density (that is, marmot density > 3 (1 marmot/hectare)).

#### Flea index

The estimates of flea burden represent fleas of all species (more than one *Xenopsylla* species can be involved in the transmission of plague). For each captured animal, the number of fleas was counted and recorded independently. The flea index of the year was calculated as the total number of collected fleas divided by the total number of animals captured in the year. We removed outliers that were outside three standard deviations of the average of the flea index (that is, flea index > 15).

#### Temperature and precipitation data

We obtained the monthly mean gridded land surface temperature and precipitation data with 0.5 × 0.5 decimal degree resolution from the Climatic Research Unit Global Climate Dataset TS4.04 released by the University of East Anglia (https://www.cru.uea.ac.uk/data) and took the average value of all grids within each county as the monthly temperature and precipitation data. The average temperature over 12 months was taken as the annual temperature of the county, and the total value of the precipitation over 12 months as the cumulative annual precipitation.

### Statistical modeling

To estimate the effects of predictor variables on the risk of marmot plague prevalence, we utilized a GAM of the binomial family to account for the nonlinear effects of various predictors on plague occurrence data. The package *mgcv* and a thin-plate spline in the R statistical programming environment (version 4.1.2) were used for these analyses, which allowed factor coefficients to vary over the values within their distributions. Candidate models included the following biological and environmental predictors: marmot density (MD), flea index (FI), annual cumulative precipitation (mm) (Pre), and average annual temperature in Celsius (Tmp). Inspection of pairwise and multivariate associations between predictor variables in the final model did not reveal serious identification problems. We estimated a set of GAM and threshold GAM formulations that modeled the various covariates, ranging from the simplest model only including time and space elements to the models including all covariates, shown in Table S1. Model selection was based on minimizing the cross-validation (CV) and Akaike's information criterion (AIC); a model with lower CV and AIC was hence preferred to those with higher values. The model CV is the average of all the squared predictive errors obtained by repeating the process of calculating a squared distance between the model prediction and the new observation for as many data points as there are observations in the original dataset. The advantage of CV compared with generalized cross-validation (GCV) is that CV can measure the effects of different models by comparing their predictive capabilities.

The final model we obtained is described by Formula 1:

1 $${{Y}}_{{{i,t}}} {{ = a}} + {{f}}_{1} \left( {{{MD}}_{i,t} } \right) + {{f}}_{2} \left( {{{FI}}_{{{i,t}}} } \right) + {{f}}_{3} \left( {{{Lon}}_{{\text{i}}} ,{{Lat}}_{{{i}}} ,{{Tmp}}_{{{i,t}}} } \right) + \begin{cases} {{f}}_{4} \left( {{{Pre}}_{{\text{i,t}}} } \right) \\ {{f}}_{5} \left( {{{Pre}}_{{{i,t}}} } \right) \end{cases} + \varepsilon_{{{i,t}}} ,\frac{{{{if\;MD}}_{{{i,t}}} < {{th}}}}{{{{Otherwise}}}}$$

where *i* = sites, *t* = time (year), and ${Y}_{i,t}$ is the logit binomial plague occurrence at site *i* in year *t*. Parameter *a* is the overall intercept, *f* is the thin-plate spline function, and $\varepsilon$ is the uncorrelated random error term.${f}_{1}\left({MD}_{i,t}\right)$ is the smooth function of marmot density at site *i* in year *t*, with a maximum of 3 *d.f.*; ${f}_{2}\left({FI}_{i,t}\right)$ is the smooth function of the flea index at site *i* in year *t*, with a maximum of 3 *d.f*.; ${f}_{3}\left({Lon}_{i},{Lat}_{i},{Tmp}_{i,t}\right)$ is a 3D tensor smooth function of the geographical location and the average annual temperature (Celsius) at site *i* in year *t*, with a maximum of 5 *d.f.* for location and 4 *d.f*. for temperature; ${f}_{4}\left({Pre}_{i,t}\right)$ is the smoothing function of annual cumulative precipitation (mm) when MD is less than the threshold value, with a maximum of 4 *d.f*.; and ${f}_{5}\left({Pre}_{i,t}\right)$ is the effect of precipitation when MD is greater than the threshold, with a maximum of 4 *d.f*..

We supposed that the impact of climatic covariates on the risk of plague occurrence differed with the density of local marmots and hence included marmot density as a threshold effect, represented by *th* in the model. The threshold value was searched for by performing a search grid throughout the entire range of the marmot density covariate and selecting the threshold that produced the best model, which is to say the one that minimized the GCV score. We also obtained threshold values for precipitation (Model 6 in Table S1) and temperature (Formula S1 in *Supplementary Information*). Because there was potential for an interaction of geographical variation and marmot density thresholds, the MD threshold value was obtained using models without the complex tensor product anisotropic function.

We also assumed that the impact of climatic covariates on risk of plague occurrence differed with location, since Xu et al.^55^ found that there was considerable heterogeneity in the effects of climate on human plague within northern and southern China and that the general effects described above are caused by changes in the extent of these areas. Thus, we used a GAM that allowed for spatially variable and nonlinear effects of climate when estimating this pattern. The geographic location-dependent effect of temperature on marmot plague was explored under a series of models (see Table S1), and the density-dependent effect of precipitation on marmot plague was explored using Formula S2 in the *Supplementary Information*.

We also investigated the effects of previous years’ and specific months’ climatic conditions on plague, which can be found in the *Supplementary Information* and Table S2. To test robustness and avoid model overfitting, shape restrictions were added to our final model. The descriptive text of the shape restriction model can be found in the *Supplementary Information,* and the results are given in Table S4 and Fig. S4.

### Assessing the effects of temperature on plague prevalence

Our study area includes all regions where marmots exist in China and Mongolia; the area is vast, and the latitude and longitude differences are enormous. Thus, geographical heterogeneity will inevitably affect the response of the plague to climate. To test the effect of geographical variation in temperature on plague prevalence, we included in the model tensor product functions of latitude, longitude, and temperature; these are embodied in Formula 1 as ${f}_{3}\left({Lon}_{i},{Lat}_{i},{Tmp}_{i,t}\right)$, provided in the *Supplementary Information*. Incorporating these functions enabled us to explore more in detail how the effects of temperature and precipitation depend on location.

The instantaneous rate of change in plague prevalence per unit change in the particular temperature covariate was measured as the partial derivative of the prevalence concerning temperature. To obtain this partial derivative, we first used the prediction function in the GAM to obtain the partial effect logit (*Y*_*tmp*_) of the local temperature at every site and year while holding other covariates fixed (marmot density, flea index, and precipitation). The vis.gam function in the *mgcv* package was used to produce contour plot views of GAM model predictions under various temperature conditions (Fig. S2).

Since our model specified that the mean prevalence rate was a nonlinear function of the covariates with a logistic link function, the probability of plague appearance *Y*_*tmp*_ was calculated using an inverse logit function, which transformed the fit value (logit (*Y*_*tmp*_)) from the predicted process to a plague prevalence value between 0 and 1. To demonstrate more local variation in the element of temperature, we obtained multiyear average predictions at each monitoring site by calculating the mean of Y_tmp_ values over all years at each point, thereby eliminating the difference in time (Fig. S3). The details of the generation process can be found in the *Supplementary Information.*

Our research area includes the QTP, a zone with an exceptional topography and climate type. To measure the difference in temperature effect between this Third Pole area and other areas, we divided the obtained *Y*_*tmp*_ according to latitude and longitude into the respective plague occurrence *Y*_*tmp1*_ in QTP and *Y*_*tmp2*_ in nQTP regions using the exact function in ArcGIS. The final partial derivative *k*_*1*_ in the QTP area and *k*_*2*_ in nQTP areas comprised the regression parameters between *Y*_*tmp1*_, *Y*_*tmp2,*_ and the temperature covariate of the corresponding region in the raw data.

### Supplementary Information

Supplementary Information.

## Data Availability

The datasets generated during and/or analyzed during the current study are available from the corresponding author upon reasonable request.

## References

[1] Gage KL, Kosoy MY (2005). Natural history of plague: Perspectives from more than a century of research. Annu. Rev. Entomol..

[2] Perry RD, Fetherston JD (1997). Yersinia pestis–etiologic agent of plague. Clin. Microbiol. Rev..

[3] Dennis DT (1999). Plague Manual: Epidemiology, Distribution, Surveillance and Control.

[4] Stenseth NC (2008). Plague: Past, present, and future. PLoS Med..

[5] Bevins SN, Baroch JA, Nolte DL, Zhang M, He H (2012). Yersinia pestis: Examining wildlife plague surveillance in China and the USA. Integr. Zool..

[6] Bertherat E, Bertherat É (2019). Plague around the world in 2019. Wkly. Epidemiol. Rec..

[7] Randremanana R (2019). Epidemiological characteristics of an urban plague epidemic in Madagascar, August–November, 2017: An outbreak report. Lancet. Infect. Dis..

[8] Hinckley A, Biggerstaff B, Griffith K, Mead P (2012). Transmission dynamics of primary pneumonic plague in the USA. Epidemiol. Infect..

[9] Balakhonov, S. *et al.* A case of human infection with plague in the Kosh-Agach region of the Republic of Altai in 2015. Communication 1. Clinical-epidemiological and epizootiological aspects. In *Problems of Particularly Dangerous Infections*, 55–60 (2016).

[10] Vallès X (2020). Human plague: An old scourge that needs new answers. PLoS Negl. Trop. Dis..

[11] Shen X, Li W (2021). Prevention and Control of Infectious Diseases in BRI Countries.

[12] Meyer KF (1942). The ecology of plague. Medicine.

[13] Wu L (1926). A Treatise on Pneumonic Plague.

[14] Wu, L.-T., Chun, J., Pollitzer, R. & Wu, C. Plague: A manual for medical and public health workers. In *Plague: A Manual for Medical and Public Health Workers.* (1936).

[15] Pollitzer R (1953). Plague studies: 8. Clinical aspects. Bull. World Health Organ..

[16] Kool JL, Weinstein RA (2005). Risk of person-to-person transmission of pneumonic plague. Clin. Infect. Dis..

[17] Mead P (2019). Epidemics of plague past, present, and future. Lancet. Infect. Dis.

[18] Kausrud K (2007). Climatically driven synchrony of gerbil populations allows large-scale plague outbreaks. Proc. R. Soc. B Biol. Sci..

[19] Snäll T, O’hara R, Ray C, Collinge S (2008). Climate-driven spatial dynamics of plague among prairie dog colonies. Am. Nat..

[20] Xu L (2015). The trophic responses of two different rodent–vector–plague systems to climate change. Proc. R. Soc. B Biol. Sci..

[21] Stenseth NC (2006). Plague dynamics are driven by climate variation. Proc. Natl. Acad. Sci..

[22] Eads DA, Hoogland JL (2017). Precipitation, climate change, and parasitism of prairie dogs by fleas that transmit plague. J. Parasitol..

[23] Cavanaugh DC (1968). Some observations on the current plague outbreak in the Republic of Vietnam. Am. J. Public Health Nations Health.

[24] Cavanaugh DC, Marshall J (1972). The influence of climate on the seasonal prevalence of plague in the Republic of Vietnam. J. Wildl. Dis..

[25] Lu L (2016). Niche modeling predictions of the potential distribution of *Marmota himalayana*, the host animal of plague in Yushu County of Qinghai. BMC Public Health.

[26] Gao M (2010). Spatial prediction and analysis of Himalayan marmot plague natural epidemic foci in China based on HJ-1 satellite data. Sci. China Earth Sci..

[27] Xu L (2019). Historical and genomic data reveal the influencing factors on global transmission velocity of plague during the Third Pandemic. Proc. Natl. Acad. Sci..

[28] Townsend SE (2009). Estimating Siberian marmot (*Marmota sibirica*) densities in the Eastern Steppe of Mongolia. Ethol. Ecol. Evol..

[29] Galdan B, Baatar U, Molotov B, Dashdavaa O (2010). Plague in Mongolia. Vector-Borne Zoonotic Dis..

[30] Ebright JR, Altantsetseg T, Oyungerel R (2003). Emerging infectious diseases in Mongolia. Emerg. Infect. Dis..

[31] Riehm JM (2011). Yersinia pestis in small rodents, Mongolia. Emerg. Infect. Dis..

[32] Kehrmann J (2020). Two fatal cases of plague after consumption of raw marmot organs. Emerg. Microbes Infect..

[33] Chen H (2022). Carbon and nitrogen cycling on the Qinghai-Tibetan Plateau. Nature Rev. Earth Environ..

[34] Liu X, Chen B (2000). Climatic warming in the Tibetan Plateau during recent decades. Int. J. Climatol. J. R. Meteorol. Soc..

[35] Wang X (2017). Mechanism study on a plague outbreak driven by the construction of a large reservoir in southwest china (surveillance from 2000–2015). PLoS Negl. Trop. Dis..

[36] Wang L-E, Zeng Y, Zhong L (2017). Impact of climate change on tourism on the Qinghai-Tibetan Plateau: Research based on a literature review. Sustainability.

[37] Suntsov V, Suntsova N (2009). Principles of speciation of the plague causative agent *Versinia pestis*: Gradualism or saltation?. Biol. Bull..

[38] Jones SD (2019). Living with plague: Lessons from the Soviet Union’s antiplague system. Proc. Natl. Acad. Sci..

[39] Eisen RJ (2020). An evaluation of the flea index as a predictor of plague epizootics in the West Nile region of Uganda. J. Med. Entomol..

[40] Biggins DE, Eads DA, Godbey JL (2021). Plague transforms positive effects of precipitation on prairie dogs to negative effects. Int. J. Parasitol. Parasites Wildl..

[41] Roberts SL, Van Wagtendonk JW, Miles AK, Kelt DA, Lutz JA (2008). Modeling the effects of fire severity and spatial complexity on small mammals in Yosemite National Park, California. Fire Ecol..

[42] Dickman C, Mahon P, Masters P, Gibson D (1999). Long-term dynamics of rodent populations in arid Australia: The influence of rainfall. Wildl. Res..

[43] Gage KL, Burkot TR, Eisen RJ, Hayes EB (2008). Climate and vectorborne diseases. Am. J. Prev. Med..

[44] Davis S (2004). Predictive thresholds for plague in Kazakhstan. Science.

[45] Davis S, Trapman P, Leirs H, Begon M, Heesterbeek J (2008). The abundance threshold for plague as a critical percolation phenomenon. Nature.

[46] Samia NI (2011). Dynamics of the plague–wildlife–human system in Central Asia are controlled by two epidemiological thresholds. Proc. Natl. Acad. Sci..

[47] Reijniers J (2012). A curve of thresholds governs plague epizootics in Central Asia. Ecol. Lett..

[48] Reijniers J, Begon M, Ageyev VS, Leirs H (2014). Plague epizootic cycles in Central Asia. Biol. Let..

[49] Brubaker, R. R. The genus yersinia: Biochemistry and genetics of virulence with 3 figures. In *Current Topics in Microbiology and Immunology: Ergebnisse der Mikrobiologie und Immunitätsforschung Volume 57*, 111-158 (1972) 10.1007/978-3-642-65297-4_4 4554354

[50] Koirala J (2006). Plague: disease, management, and recognition of act of terrorism. Infect. Dis. Clin..

[51] Nozadze M (2015). Comparative proteomic studies of *Yersinia pestis* strains isolated from natural Foci in the Republic of Georgia. Front. Public Health.

[52] Krasnov B, Khokhlova I, Fielden L, Burdelova N (2001). Development rates of two *Xenopsylla flea* species in relation to air temperature and humidity. Med. Vet. Entomol..

[53] Krasnov B, Burdelova N, Shenbrot G, Khokhlova I (2002). Annual cycles of four flea species in the central Negev desert. Med. Vet. Entomol..

[54] He Z (2021). Distribution and characteristics of human plague cases and *Yersinia pestis* isolates from 4 *marmota* plague foci, China, 1950–2019. Emerg. Infect. Dis..

[55] Xu L (2011). Nonlinear effect of climate on plague during the third pandemic in China. Proc. Natl. Acad. Sci..

[56] Fang X, Zhou D (2015). The evolutionary dynamics and the ecological niche of natural plague foci in China. Chin. J. Vector Biol. Control..

[57] Brandler OV (2021). A study of hybridization between *Marmota baibacina* and *M. sibirica* in their secondary contact zone in Mongolian Altai. Front. Ecol. Evol..

[58] Pisarenko SV (2021). *Yersinia pestis* strains isolated in natural plague foci of Caucasus and Transcaucasia in the context of the global evolution of species. Genomics.

