# Author Correction: Climate-driven marmot-plague dynamics in Mongolia and China

**DOI:** 10.1038/s41598-023-41800-3

**Published:** 2023-09-05

**Authors:** Lei Xu, Qian Wang, Ruifu Yang, Dalantai Ganbold, Nyamdorj Tsogbadrakh, Kaixing Dong, Min Liu, Doniddemberel Altantogtokh, Qiyong Liu, Sainbileg Undrakhbold, Bazartseren Boldgiv, Wannian Liang, Nils Chr. Stenseth

**Affiliations:** 1 Vanke School of Public Health, Tsinghua University, Beijing, 100084 China; 2 Beijing Institute of Microbiology and Epidemiology, Beijing, 100071 China; 3 National Center for Zoonotic Diseases, Ulaanbaatar, 211137 Mongolia; 4 Department of Epidemiology and Biostatistics, School of Public Health, Peking University, Beijing, 100191 China; 5 State Key Laboratory of Infectious Disease Prevention and Control, National Institute for Communicable Disease Control and Prevention, Chinese Center for Disease Control and Prevention, Changping, Beijing, 102206 China; 6 Professional Biological Society of Mongolia, Ulaanbaatar, 14201 Mongolia; 7 Department of Biology, National University of Mongolia, Ulaanbaatar, 14201 Mongolia; 8 The Centre for Pandemics and One‑Health Research, Faculty of Medicine, University of Oslo, Oslo, Norway; 9 Centre for Ecological and Evolutionary Synthesis, Department of Biosciences, Faculty of Mathematics and Natural Sciences, University of Oslo, Oslo, Norway

Correction to: *Scientific Reports* https://doi.org/10.1038/s41598-023-38966-1, published online 24 July 2023

The original version of this Article contained a formatting error in Equation 1. As a result, the subsequent paragraph,

*“Y*_*it*_ is the logit binomial plague occurrence at site *i* in year *t*. Parameter *a* is the overall intercept, *f* is the thin-plate spline function, and ε is the uncorrelated random error term. $f$_1_(*RD*_*i,t*_) is the smooth function of marmot density at site *i* in year *t,”*

now reads:

*“Y*_*i,t*_ is the logit binomial plague occurrence at site *i* in year *t*. Parameter *a* is the overall intercept, *f* is the thin-plate spline function, and ε is the uncorrelated random error term. $f$_1_(*MD*_*i,t*_) is the smooth function of marmot density at site *i* in year *t,”*

The original Article has been corrected.

