# Supplementary material for: Climate-driven marmot-plague dynamics in Mongolia and China
Source: Sci Rep. 2023 Jul 24;13:11906. doi: 10.1038/s41598-023-38966-1 (PMC10366125; doi:10.1038/s41598-023-38966-1)
Supplement: Supplementary file 1 — Supplementary Information. [file 41598_2023_38966_MOESM1_ESM.docx]

**Supplementary Information**

**Climate-driven marmot-plague dynamics in Mongolia and China**

Lei Xu^a^, Qian Wang^a^, Ruifu Yang^b^, Dalantai Ganbold^c^, Nyamdorj Tsogbadrakh^c^, Kaixing Dong^a^, Min Liu^d^, Doniddemberel Altantogtokh^c^, Qiyong Liu^e^, Sainbileg Undrakhbold^f,g^, Bazartseren Boldgiv^g,^*, Wannian Liang ^a,^*, Nils Chr. Stenseth^h,i,^*

^a^ Vanke school of public health, Tsinghua University, Beijing, 100084, China

^b^ Beijing Institute of Microbiology and Epidemiology, Beijing, 100071, China

^c^ National Center for Zoonotic Diseases, Ulaanbaatar, 211137, Mongolia

^d^ Department of Epidemiology and Biostatistics, School of Public Health, Peking University, Beijing 100191, China

^e^ State Key Laboratory of Infectious Disease Prevention and Control, National Institute for Communicable Disease Control and Prevention, Chinese Center for Disease Control and Prevention, Changping, Beijing 102206, China

^f^ Professional Biological Society of Mongolia, Ulaanbaatar, 14201, Mongolia

^g^ Department of Biology, National University of Mongolia, Ulaanbaatar, 14201, Mongolia

^h^The Centre for Pandemics and One-Health Research, Faculty of Medicine, University of Oslo, Oslo, Norway.

^i^ Centre for Ecological and Evolutionary Synthesis, Department of Biosciences, Faculty of Mathematics and Natural Sciences, University of Oslo, N-0316 Oslo, Norway

*Corresponding authors:

**Email:**

[boldgiv@num.edu.mn](mailto:boldgiv@num.edu.mn) (B.B.);

[liangwn@tsinghua.edu.cn](mailto:liangwn@tsinghua.edu.cn) (WN.L.);

[n.c.stenseth@mn.uio.no](mailto:n.c.stenseth@mn.uio.no) (N.C.S)

Supplementary text

Figures S1 to S4

Tables S1 to S4

**Supplementary Information Text**

**Effect visualization of Fig. 3 in main text**

To get the total range value of the plague epidemic interpolation map covering China and Mongolia, we first obtain the average value $\underline{Y}_{tmp}$ of each point by recalculating the average of all year values of *Y_tmp_* value at each point to eliminate the difference in time. In this way, $\underline{Y}_{tmp}$ only differs in the spatial dimension, and we presented it in Fig. 3 through its corresponding latitude and longitude and marked the value difference via the size of the redpoint.

Then the Inverse Distance Weighted (IDW) method was used to predict values for unsampled location by using the measured values surrounding the prediction locations. IDW is based on the assumption that things close to one another are more alike than those farther apart. Based on the consideration of IDW that each measured point has a local influence that diminishes with distance, it gives greater weights to points closest to the prediction location, and the weights diminish as a function of distance, hence the name inverse distance is weighted. We use color gradient changes (yellow to grey to red) to show the plague prevalence of the entire study area under the geographical heterogeneity of temperature influence after the final interpolation.

**Cross-checking of two special properties on temperature and precipitation**

Considering that biological factors may have different constraints on the impact of different environmental factors on the plague, we also checked the temperature constraints of marmot density and constructed the following model:

$Y_{i,t}=a+f_{1}\left( {FI}_{i,t} \right)+f_{2}\left( {Pre}_{i,t} \right)+\{f_{3}\left( {Tmp}_{i,t} \right) f_{4}\left( {Tmp}_{i,t} \right) {+\varepsilon}_{it},\frac{if {MD}_{i,t}<th}{Otherwise}$ (S1)

where *i* = sites, *t*=time(year), $Y_{i,t}$ is the logit binomial plague occurrence in site *i* and in the year *t*. Parameter *a* is the overall intercept, *f* are thin-plate spline functions, and $\varepsilon$ is the uncorrelated random error term.$f_{1}\left( {FI}_{i,t} \right)$ is the smooth function of flea index in site *i* and in the year *t*. $f_{2}\left( {Pre}_{i,t} \right)$ is the smooth function of precipitation in site *i* and in the year *t*. $f_{3}\left( {Tmp}_{i,t} \right)$ is the smooth function of temperature when MD is less than threshold value and $f_{4}\left( {Tmp}_{i,t} \right)$ is the effect of temperature when MD is more than the threshold. There is threshold effect of marmot density on temperature, as shown in Table S3.

For the geographical heterogeneity of the plague in temperature as Figs. 3 and 4, we also tested it on precipitation and established a model as below.

$Y_{i,t}=a+f_{1}\left( {MD}_{i,t} \right)+f_{2}\left( {FI}_{i,t} \right)+f_{3}\left( {Lon}_{i},{Lat}_{i},{Pre}_{i,t} \right)+f_{4}\left( {Tmp}_{i,t} \right){+\varepsilon}_{i,t}$ (S2)

Where *i* = sites, *t*=time(year), $Y_{i,t}$ is the logit binomial plague occurrence in site *i* and in the year *t*. Parameter *a* is the overall intercept, *f* are thin-plate spline functions, and $\varepsilon$ is the uncorrelated random error term.$f_{1}\left( {MD}_{i,t} \right)$ is the smooth function of marmot density in site *i* and in the year *t*. $f_{2}\left( {FI}_{i,t} \right)$ is the smooth function of flea index in site *i* and in the year *t*. $f_{3}\left( {Lon}_{i},{Lat}_{i},{Pre}_{i,t} \right)$ is a 3D tensor smooth function of the geographical location and of the annual cumulative precipitation (mm) in year *t* at site *i* (with maximally 5 *d.f.* for location and 4 *d.f.* for precipitation). $f_{4}\left( {Tmp}_{i,t} \right)$ is the smooth function of annual average temperature in site *i* and in the year *t*. And we did not find a significant effect of $f_{3}\left( {Lon}_{i},{Lat}_{i},{Pre}_{i,t} \right)$ on marmot plague thus our result indicates there is no spatial heterogeneity effect when it comes to precipitation, as shown in Table S3.

**Time-lag test and season test of effect of precipitation and temperature on marmot plague**

In order to test whether the influence of climate factors on the occurrence of plague has a time lag effect and season effect, all the models which include temperature and precipitation in Table S1 have been performed more times by replacing with the temperature and precipitation of the previous year and with the temperature and precipitation of specific months’ data for verification, as shown in Table. S2. Based on the marmot hibernation time and the timing of field monitoring in our study area (1), we used meteorological data of two specific periods (from May to September and March to October) of each year as the seasonal pattern test.

When the climatic elements of the previous year and current year’s specific months are considered together, based on cross-validation (CV) value, which is an indicator for comparing simulation performance across multiple models 11 (CV: 0.3218) in Table S1 has better performance in the real situation of simulated data than all the models with previous year’s and specific months’ climatic variables in Table S2. In view of the fact that the impact of the climate across the years and that of the current year on animals will change over time, and our model results show that the simulation of current year climate on marmot plague are still the best with the lowest CV, so in this study, only the current year’s climatic factors will be discussed and the complicated relationship between current year’s temperature and precipitation and the occurrence of marmot, flea index and marmot plague will be explored in-depth.

**Shape restrictions on biological covariates**

We added shape restrictions on biological covariates (FI and MD) to see if there was overfitting in our final model. The SCAM package in R was used to avoid model overfit. Based on the previous research background on the relationship between marmot density and flea index and prevalence of plague, we added the positive shape restrictions both on MD and FI with bs = “mpi” in 11 as formula (S3). Bs= “mpi” is a special method function for creating smooths subject to a monotone increasing constraint which is built by the mgcv constructor function for smooth terms. The partial effects on the plague prevalence of the marmot population density, flea index, and annual cumulative precipitation were given in Fig. S4 and the result summary could be found in Table S4.

$Y_{i,t}=f_{1}\left( {MD}_{i,t}, bs="mpi" \right)+f_{2}\left( {FI}_{i,t}, bs="mpi" \right)+f_{3}\left( {Lon}_{i},{Lat}_{i},{Tmp}_{i,t} \right)+\{f_{4}\left( {Pre}_{i,t} \right) f_{5}\left( {Pre}_{i,t} \right) +\varepsilon_{i,t},\frac{if MD <th}{Otherwise}$ (S3)

Based on the statistical model result, we found that marmot plague occurrence exhibited a significant upward trend with the flea index (*P* < *0.05*; see Fig. S4B), which indicated that the risk of plague occurrence increased with the increases of the flea index. In terms of the effect of precipitation, the shape restrictions model showed its significance on the marmot plague (see Table S4). However, after considering the nonadditive probability between precipitation and marmot density, we utilized marmot density as a threshold for judging the impact of precipitation on the occurrence of plague. Different interaction curves were found between precipitation and plague when the marmot density was in two different states (see Figs.S4 C and D): when the marmot density was lower than 0.63 (1 marmot/1 hectare), the risk of plague change with the amount of precipitation (*P* < *0.05*; see Fig. S4C); when the marmot density was higher than 0.63 (1 marmot/1 hectare), high rainfall increased the risk of plague (*P* < *0.01*; see Fig. S4D). And the synergistic effect of temperature and latitude and longitude still showed significance (*P* < *0.05*; see Table S4) under shape restrictions on biological covariates.


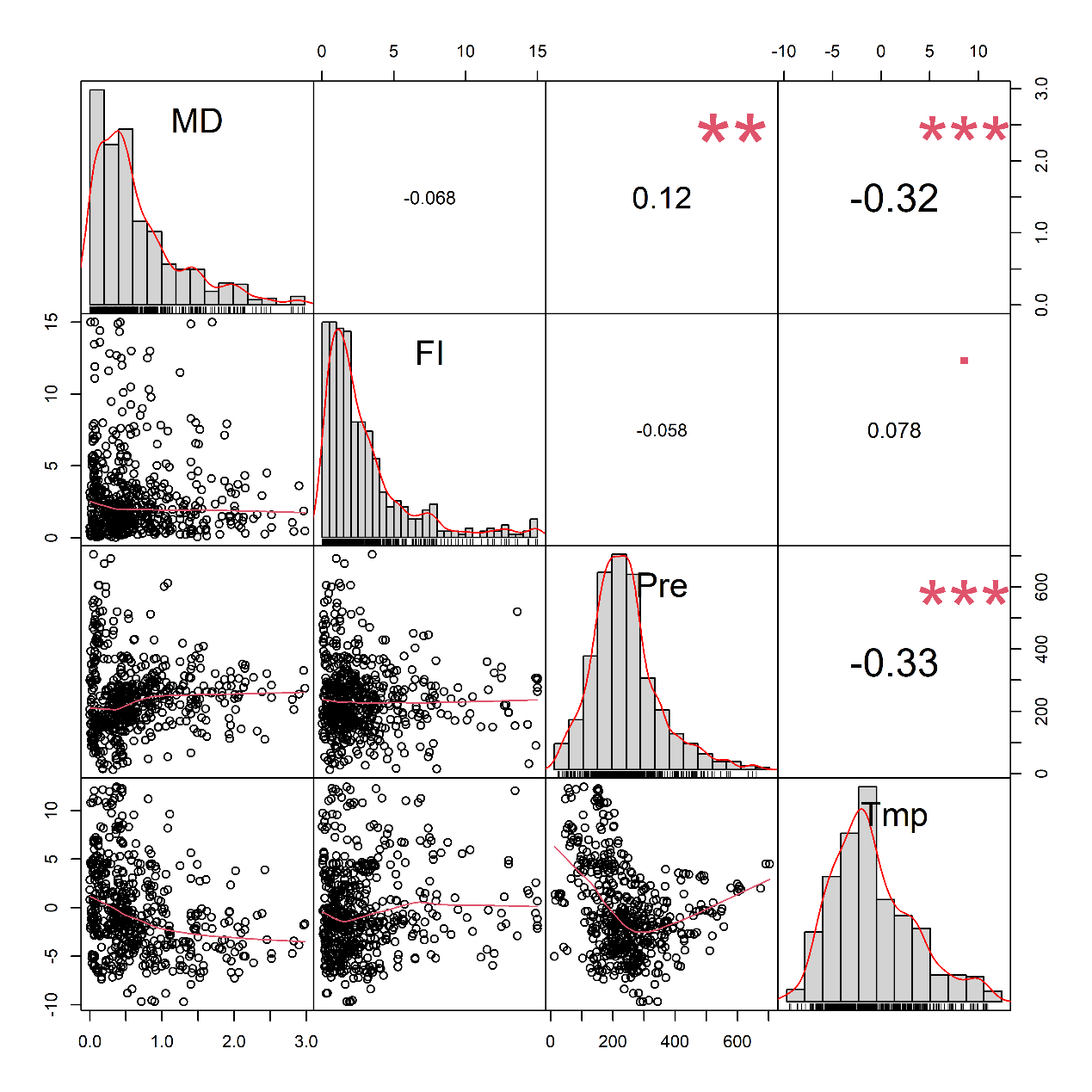


**Fig. S1.** The result of the Spearman test for covariates. This indicates that marmot density (MD), flea index (FI), annual cumulative precipitation (mm) (Pre), and annual average temperature (Celsius) (Tmp) have no strong collinearity, and all factors can be selected into the regression model.

**
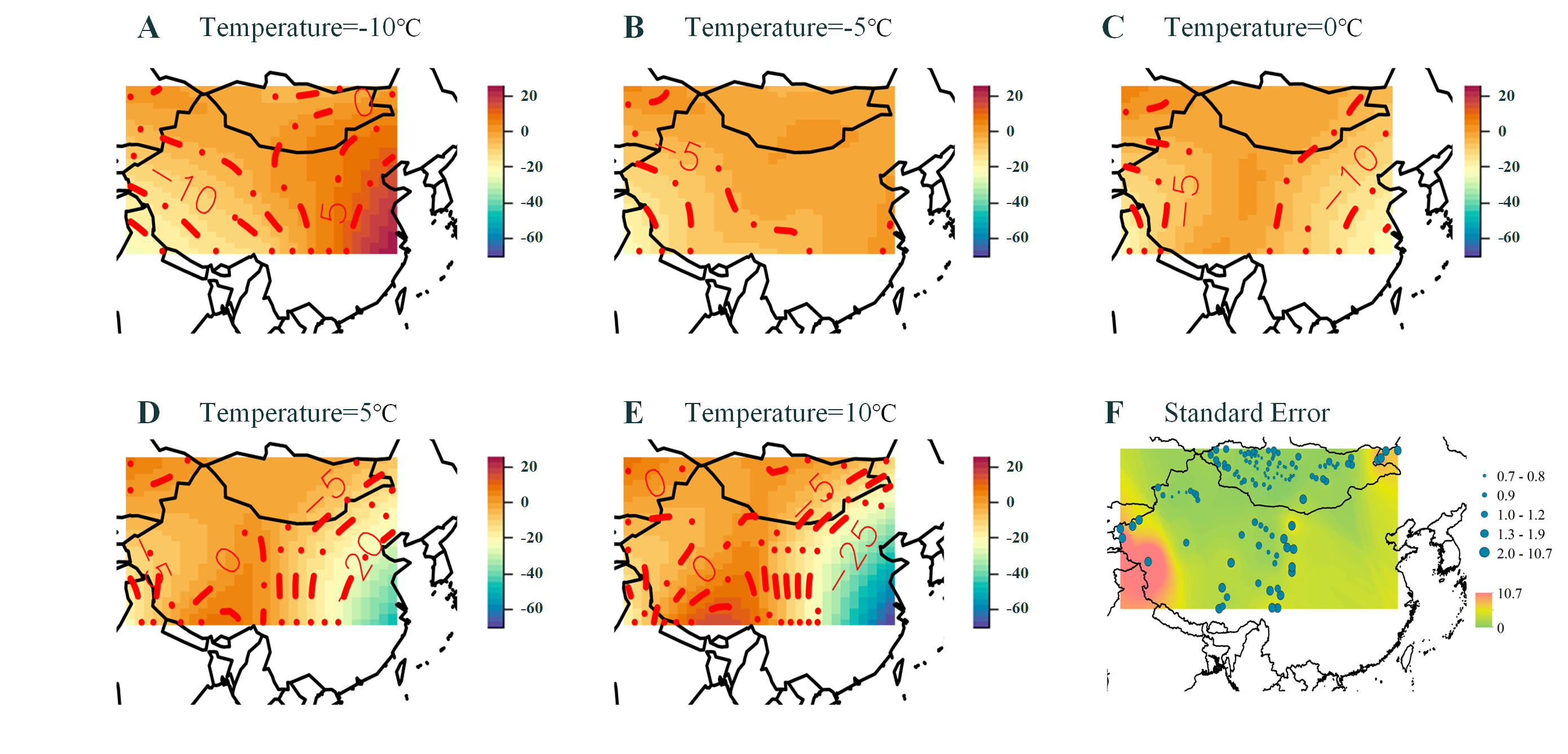
**

**Fig. S2**. Predicted plague of marmot plague cases from final model covering both Mongolia and China under extremely cold, very cold, normal, hot and very hot conditions (A–E) and the standard error of temperature (F). The color gradient of A-E represents predicted temperature partial effect on plague ranging from blue (low: -68.7) to red (high:23.6). The color gradient of F represents standard error value from light green (low:0) to red (high:10.7).


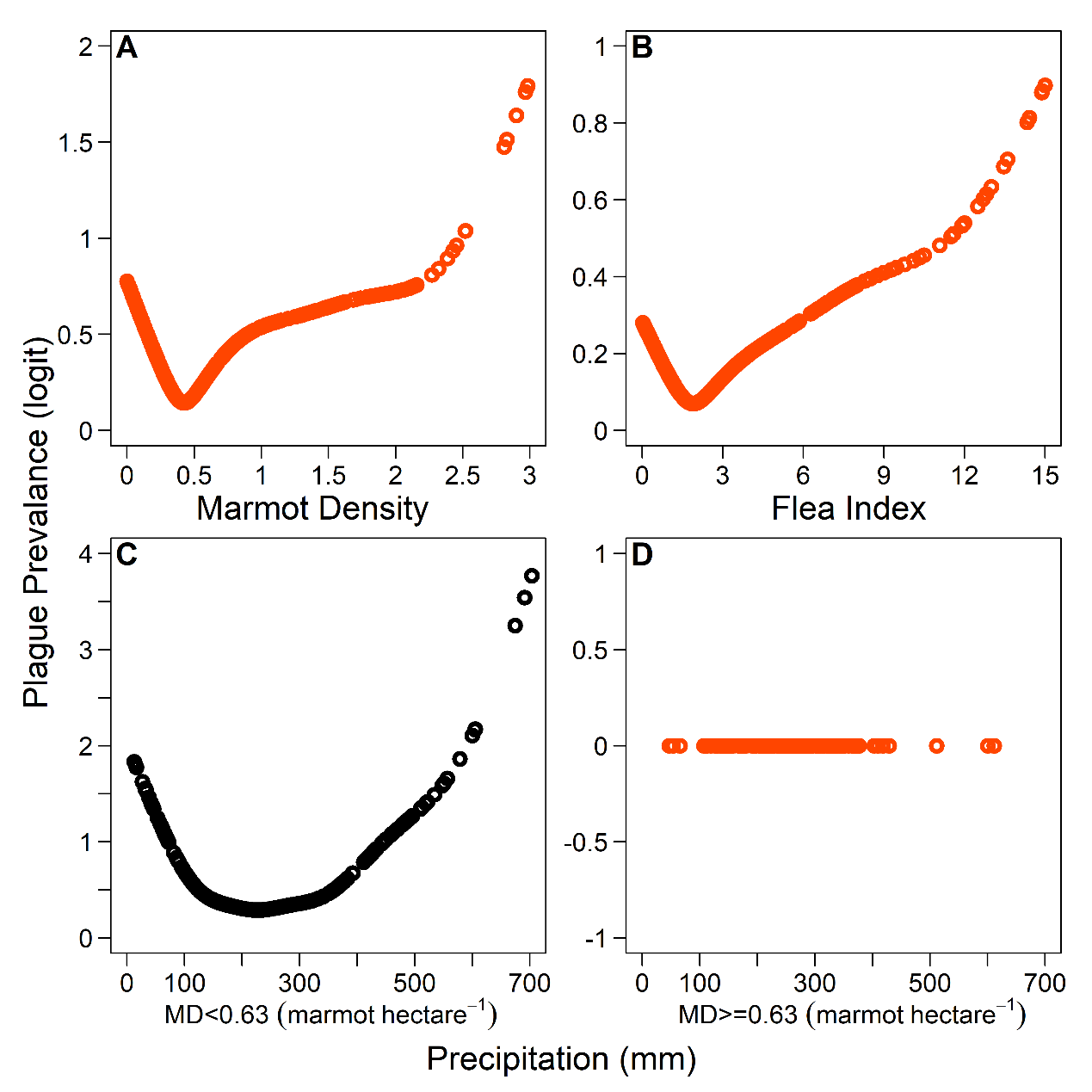


**Fig. S3.** The standard error obtained from formula 1 of A. marmot density; B. flea index; C. precipitation when marmot density < 0.63; and D. precipitation when marmot density >= 0.63.


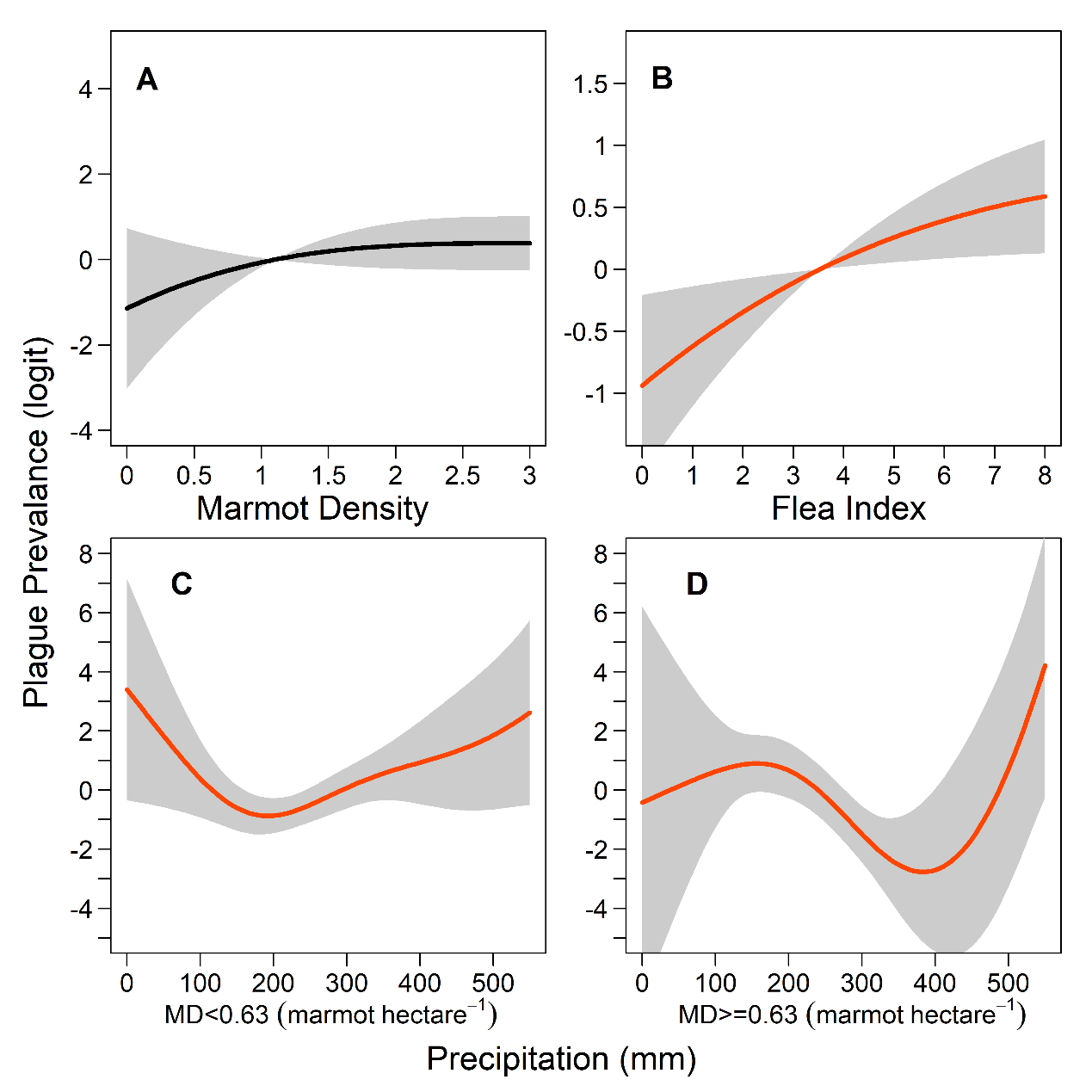


Fig. S4. The partial effects obtained from shape restrictions model 11 on the plague prevalence of A. marmot density; B. flea index; C. precipitation when marmot density < 0.63; and D. precipitation when marmot density >= 0.63.

**Table S1.** Model selection using CV and delta-AIC**.** Models with lower CV values have higher out-of-sample predictive power. We set the AIC value of model 11 as the benchmark. Lower AIC values indicate a better-fit model, and a model with a delta-AIC (the difference between the two AIC values being compared) of more than -2 AIC units is considered significantly better than the model it is being compared to. Model 11 is thus the final model with the lowest AIC and lowest CV.

| **Model** | **Model type** | **Formula** | **Significant**  **factors** | **delta-AIC** | **Deviance explained** | **CV** |
| --- | --- | --- | --- | --- | --- | --- |
| 1 | Geography + year | $Y_{i,t}=f_{1}\left( {lon}_{i},{lat}_{i} \right)+f_{2}\left( {year}_{i,t} \right)+\varepsilon_{i,t}$ | s(lon, lat), year | 80.3087 | 16.7% | 0.4802 |
| 2 | Biology | $Y_{i,t}=f_{1}\left( {MD}_{i,t} \right)+f_{2}\left( {FI}_{i,t} \right)+\varepsilon_{i,t}$ | FI | 114.4076 | 7.94% | 0.3602 |
| 3 | Biology + Tmp | $Y_{i,t}=f_{1}\left( {MD}_{i,t} \right)+f_{2}\left( {FI}_{i,t} \right)$*+*$f_{3}\left( {Tmp}_{i,t} \right)+\varepsilon_{i,t}$ | FI | 112.2272 | 9.93% | 0.3601 |
| 4 | Biology + Pre | $Y_{i,t}=f_{1}\left( {MD}_{i,t} \right)+f_{2}\left( {FI}_{i,t} \right)$*+*$f_{3}\left( {Pre}_{i,t} \right)+\varepsilon_{i,t}$ | FI, Pre | 74.4047 | 18% | 0.3353 |
| 5 | Biology + Tmp + Pre | $Y_{i,t}=f_{1}\left( {MD}_{i,t} \right)+f_{2}\left( {FI}_{i,t} \right)$*+*$f_{3}\left( {Tmp}_{i,t} \right)+f_{4}\left( {Pre}_{i,t} \right)+\varepsilon_{i,t}$ | FI, Pre | 74.385 | 19.6% | 0.3366 |
| 6 | Biology + Tmp + Pre by MD threshold | $Y_{i,t}=f_{1}\left( {FI}_{i,t} \right)+f_{2}\left( {Tmp}_{i,t} \right)+\{f_{3}\left( {Pre}_{i,t} \right) f_{4}\left( {Pre}_{i,t} \right) +\varepsilon_{i,t},\frac{if MD <th}{Otherwise}$ | FI, Pre | 63.7568 | 22.6% | 0.3394 |
| 7 | Biology + Pre+ Tmp  By region | $Y_{i,t}=f_{1}\left( {RD}_{i,t} \right)+f_{2}\left( {FI}_{i,t}, \right)+f_{3}\left( {Pre}_{i,t} \right)+\{f_{4}\left( {Tmp}_{i,t} \right) f_{5}\left( {Tmp}_{i,t} \right) +\varepsilon_{i,t,\frac{if in Tibetan Plateau region}{Otherwise}}$ | FI, Pre, Tmp | 45.11173 | 28.2% | 0.322 |
| 8 | Biology + geography +Tmp+ Pre by MD threshold | $Y_{i,t}=f_{1}\left( {MD}_{i,t} \right)+f_{2}\left( {FI}_{it} \right)+f_{3}\left( {{lon}_{i},lat}_{i} \right)+f_{4}\left( {Tmp}_{i,t} \right)+\{f_{5}\left( {Pre}_{i,t} \right) f_{6}\left( {Pre}_{i,t} \right) +\varepsilon_{i,t},\frac{if MD <th}{Otherwise}$ | FI, Tmp, s(lon, lat), Pre when MD < th | 44.9963 | 28.5% | 0.3221 |
| 9 | Biology + Pre + te(Tmp) | $Y_{i,t}=f_{1}\left( {MD}_{i,t} \right)+f_{2}\left( {FI}_{i,t} \right)+f_{3}\left( {Pre}_{i,t} \right)+f_{4}\left( {{lon}_{i},{lat}_{i},Tmp}_{i,t} \right){+\varepsilon}_{i,t}$ | MD, FI, te(lon, lat,Tmp) | 14.0377 | 40.5% | 0.3329 |
| 10 | FI + te(lon,lat,Tmp) + Pre by MD threshold | $Y_{i,t}=f_{1}\left( {FI}_{i,t} \right)+f_{2}\left( {{lon}_{i},{lat}_{i},Tmp}_{i,t} \right)+\{f_{3}\left( {Pre}_{i,t} \right) f_{4}\left( {Pre}_{i,t} \right) +\varepsilon_{i,t},\frac{if MD <th}{Otherwise}$ | te(lon, lat,Tmp), Pre | 4.6194 | 43.8% | 0.3335 |
| 11* | Biology + te(lon,lat,Tmp) + Pre by MD threshold | $Y_{i,t}=f_{1}\left( {MD}_{i,t} \right)+f_{2}\left( {FI}_{i,t} \right)+f_{3}\left( {{lon}_{i},{lat}_{i},Tmp}_{i,t} \right)+\{f_{4}\left( {Pre}_{i,t} \right) f_{5}\left( {Pre}_{i,t} \right) +\varepsilon_{i,t},\frac{if MD <th}{Otherwise}$ | MD, FI, te(lon, lat,Tmp)  Pre when MD > th | 0 | 46.1% | 0.3218 |

***Model 11 is the final model used in our study.**

**Table S2.** Time lag and seasonal test of the impact of temperature and precipitation on plague. All the models which include temperature and precipitation in Table S1 have been run again by replacing with the temperature and precipitation of the previous year and specific months’ data for verification. Model names with the suffix lag1 were replaced with temperature and precipitation of the previous year, and those with the suffixes (5-9) and (3-10) were replaced with temperature and precipitation from May to September and March to October of the current year.

| **Model** | **Model type** | **Formula** | **Significant factors** | **AIC** | **Deviance explained** | **CV** |
| --- | --- | --- | --- | --- | --- | --- |
| 3.lag1 | Biology + Tmp | $Y_{i,t}=f_{1}\left( {MD}_{i,t} \right)+f_{2}\left( {FI}_{i,t} \right)$*+*$f_{3}\left( {Tmp}_{i,t-1} \right)+\varepsilon_{i,t}$ | FI | 436.6242 | 10.2% | 0.3598 |
| 3(5-9) |  | $Y_{i,t}=f_{1}\left( {MD}_{i,t} \right)+f_{2}\left( {FI}_{i,t} \right)$*+*$f_{3}\left( {Tmp}_{i,t} \right)+\varepsilon_{i,t}$ | FI | 437.9538 | 9.75% | 0.3567 |
| 3(3-10) |  |  | FI | 437.1124 | 10.1% | 0.3559 |
| 4.lag1 | Biology + Pre | $Y_{i,t}=f_{1}\left( {MD}_{i,t} \right)+f_{2}\left( {FI}_{i,t} \right)$*+*$f_{3}\left( {Pre}_{i,t-1} \right)+\varepsilon_{i,t}$ | FI, Pre_it-1_ | 394.0804 | 19.5% | 0.3371 |
| 4(5-9) |  | $Y_{i,t}=f_{1}\left( {MD}_{i,t} \right)+f_{2}\left( {FI}_{i,t} \right)$*+*$f_{3}\left( {Pre}_{i,t} \right)+\varepsilon_{i,t}$ | FI, Pre | 418.7545 | 13.6% | 0.3482 |
| 4(3-10) |  |  | FI, Pre | 407.6689 | 16.4% | 0.3396 |
| 5.lag1 | Biology + Tmp + Pre | $Y_{i,t}=f_{1}\left( {MD}_{i,t} \right)+f_{2}\left( {FI}_{i,t} \right)$*+*$f_{3}\left( {Tmp}_{i,t-1} \right)+f_{4}\left( {pre}_{i,t-1} \right)+\varepsilon_{i,t}$ | FI, Pre_it-1_ | 389.0824 | 22.3% | 0.3378 |
| 5(5-9) |  | $Y_{i,t}=f_{1}\left( {MD}_{i,t} \right)+f_{2}\left( {FI}_{i,t} \right)$*+*$f_{3}\left( {Tmp}_{i,t} \right)+f_{4}\left( {Pre}_{i,t} \right)+\varepsilon_{i,t}$ | FI, Pre | 416.872 | 15.3% | 0.3478 |
| 5(3-10) |  |  | FI, Pre | 405.6587 | 18.3% | 0.3393 |
| 6.lag1 | Biology + Tmp + Pre by MD threshold | $Y_{i,t}=f_{1}\left( {FI}_{i,t} \right)+f_{2}\left( {Tmp}_{i,t-1} \right)+\{f_{3}\left( {Pre}_{i,t-1} \right) f_{4}\left( {Pre}_{i,t-1} \right) +\varepsilon_{i,t},\frac{if MD <th}{Otherwise}$ | FI, Pre_it-1_ when MD < th | 391.8519 | 21.5% | 0.3419 |
| 6(5-9) |  | $Y_{i,t}=f_{1}\left( {FI}_{i,t} \right)+f_{2}\left( {Tmp}_{i,t} \right)+\{f_{3}\left( {Pre}_{i,t} \right) f_{4}\left( {Pre}_{i,t} \right) +\varepsilon_{i,t},\frac{if MD <th}{Otherwise}$ | FI, Pre | 411.0188 | 17.7% | 0.3484 |
| 6(3-10) |  |  | FI, Pre | 396.6555 | 21% | 0.3433 |
| 7.lag1 | Biology + Pre + Tmp by location | $Y_{i,t}=f_{1}\left( {MD}_{i,t} \right)+f_{2}\left( {FI}_{i,t} \right)+f_{3}\left( {Pre}_{i,t-1} \right)+\{f_{4}\left( {Tmp}_{i,t-1} \right) f_{5}\left( {Tmp}_{i,t-1} \right) +\varepsilon_{i,t,\frac{if in Tibetan Plateau region}{Otherwise}}$ | FI, Pre_i,t-1,_ Tmp _i,t-1_ | 371.4582 | 28.1% | 0.3288 |
| 7(5-9) |  | $Y_{i,t}=f_{1}\left( {MD}_{i,t} \right)+f_{2}\left( {FI}_{i,t} \right)+f_{3}\left( {Pre}_{i,t} \right)+\{f_{4}\left( {Tmp}_{i,t} \right) f_{5}\left( {Tmp}_{i,t} \right) +\varepsilon_{i,t,\frac{if in Tibetan Plateau region}{Otherwise}}$ | FI, Pre,Tmp | 411.4148 | 18.2% | 0.3391 |
| 7(3-10) |  |  | FI, Pre, Tmp in non-Tibetan Plateau region | 391.0305 | 23.3% | 0.3316 |
| 8.lag1 | Biology +s(x,y)+ Tmp + Pre by MD threshold | $Y_{i,t}=f_{1}\left( {MD}_{i,t} \right)+f_{2}\left( {FI}_{i,t}, \right)+f_{3}\left( {{lon}_{i},{lat}_{i}} \right)+f_{4}\left( {Tmp}_{i,t-1} \right)+\{f_{5}\left( {Pre}_{i,t-1} \right) f_{6}\left( {Pre}_{i,t-1} \right) +\varepsilon_{i,t,}\frac{if MD <th}{Otherwise}$ | MD, FI, Pre _i, t-1_ when MD < th | 377.0263 | 28% | 0.3336 |
| 8(5-9) |  | $Y_{i,t}=f_{1}\left( {MD}_{i,t} \right)+f_{2}\left( {FI}_{i,t}, \right)+f_{3}\left( {{lon}_{i},{lat}_{i}} \right)+f_{4}\left( {Tmp}_{i,t} \right)+\{f_{5}\left( {Pre}_{i,t} \right) f_{6}\left( {Pre}_{i,t} \right) +\varepsilon_{i,t,}\frac{if MD <th}{Otherwise}$ | FI, s(lon, lat), Tmp, Pre_it-1_ when MD < th | 376.8311 | 28.5% | 0.3246 |
| 8(3-10) |  |  | FI, s(lon, lat), Tmp, Pre_it-1_ | 375.4023 | 27.9% | 0.3258 |
| 9.lag1 | Biology + Pre + te(lon,lat,Tmp) | $Y_{i,t}=f_{1}\left( {MD}_{i,t} \right)+f_{2}\left( {FI}_{i,t} \right)+f_{3}\left( {Pre}_{i,t-1} \right)+f_{4}\left( {{lon}_{i},{lat}_{i},Tmp}_{i,t-1} \right){+\varepsilon}_{i,t}$ | Nan | 325.5017 | 54.9% | 0.3553 |
| 9(5-9) |  | $Y_{i,t}=f_{1}\left( {MD}_{i,t} \right)+f_{2}\left( {FI}_{i,t} \right)+f_{3}\left( {Pre}_{i,t} \right)+f_{4}\left( {{lon}_{i},{lat}_{i},Tmp}_{i,t} \right){+\varepsilon}_{i,t}$ | te(lon, lat,Tmp) | 333.1498 | 46.2% | 0.3685 |
| 9(3-10) |  |  | MD, FI, te(x,y,Tmp) | 338.515 | 42.1% | 0.3233 |
| 10.lag1 | FI + te(lon,lat,Tmp) + Pre by MD threshold | $Y_{i,t}=f_{1}\left( {FI}_{i,t} \right)+f_{2}\left( {{lon}_{i},{lat}_{i},Tmp}_{i,t-1} \right)+\{f_{3}\left( {Pre}_{i,t-1} \right) f_{4}\left( {Pre}_{i,t-1} \right) +\varepsilon_{i,t,}\frac{if MD <th}{Otherwise}$ | Nan | 325.9991 | 57.7% | 0.3576 |
| 10(5-9) |  | $Y_{i,t}=f_{1}\left( {FI}_{i,t} \right)+f_{2}\left( {{lon}_{i},{lat}_{i},Tmp}_{i,t} \right)+\{f_{3}\left( {Pre}_{i,t} \right) f_{4}\left( {Pre}_{i,t} \right) +\varepsilon_{i,t,}\frac{if MD <th}{Otherwise}$ | te (lon, lat, Tmp), Pre when MD > th | 332.3929 | 45.6% | 0.3813 |
| 10(3-10) |  |  | te (lon, lat, Tmp), Pre when MD > th | 331.7059 | 45% | 0.331 |
| 11.lag1 | Biology + te(lon,lat,Tmp) + Pre by MD threshold | $Y_{i,t}=f_{1}\left( {MD}_{it} \right)+f_{2}\left( {FI}_{it} \right)+f_{3}\left( {{lon}_{i},{lat}_{i},Tmp}_{i,t-1} \right)+\{f_{4}\left( {Pre}_{i,t-1} \right) f_{5}\left( {Pre}_{i,t-1} \right) +\varepsilon_{i,t},\frac{if MD <th}{Otherwise}$ | Nan | 321.4868 | 59.7% | 0.3436 |
| 11(5-9) |  | $Y_{i,t}=f_{1}\left( {MD}_{i,t} \right)+f_{2}\left( {FI}_{i,t} \right)+f_{3}\left( {{lon}_{i},{lat}_{i},Tmp}_{i,t} \right)+\{f_{4}\left( {Pre}_{i,t} \right) f_{5}\left( {Pre}_{i,t} \right) +\varepsilon_{i,t},\frac{if MD <th}{Otherwise}$ | MD, FI, te(lon, lat,Tmp) ， Pre when MD > th | 326.08 | 47.9% | 0.365 |
| 11(3-10) |  |  | MD, FI, te(lon, lat,Tmp)， Pre when MD > th | 324.8457 | 46.6% | 0.3249 |

**Table S3.** Approximate significance of smooth terms in formula s1 and s2.

| **Effect in formula. s1** | | | **Effect in formula. s2** | | |
| --- | --- | --- | --- | --- | --- |
| Factors | edf | P-value | Factors | edf | P-value |
| *s(FI)* | 2.062 | *0.00139*** | *s(MD)* | 1.986 | 0.3765 |
| *s(Pre)* | 3.544 | *<0.001**** | *s(FI)* | 2.075 | 0.7030 |
| *s(Tmp)* (when MD < th) | 3.494 | *0.04926** | *s(Tmp)* | 1.0 | 0.0792 |
| *s(Tmp)* (when MD >= th) | 3.933 | *0.02015** | *te(lat,lon,Pre)* | 88.682 | 1.0 |

Signif. codes: 0 ‘***’ 0.001 ‘**’ 0.01 ‘*’ 0.05 ‘.’ 0.1 ‘ ’ 1

**Table S4.** Results of shape restrictions model 11 as formula (s3), including the edf and p-values of every term, the deviance explained and AIC.

| **Factors** | **edf** | **P-value** |
| --- | --- | --- |
| *s(MD*, bs= “mpi”*)* | 1 | 0.2236 |
| *s(FI*, bs= “mpi”*)* | 1 | 0.0103* |
| *s(Pre)* (when MD < th) | 3.309 | 0.0111* |
| *s(Pre)* (when MD >= th) | 3.684 | 0.0431* |
| *te(x,y,tmp)* | 22.596 | 0.0040** |
| *Deviance explained* | 44.1% | |
| *AIC* | 329.2187 | |

Signif. codes: 0 ‘***’ 0.001 ‘**’ 0.01 ‘*’ 0.05 ‘.’ 0.1 ‘ ’ 1

**Reference:**

1. Z. He *et al.*, Distribution and Characteristics of Human Plague Cases and Yersinia pestis Isolates from 4 Marmota Plague Foci, China, 1950–2019. *Emerg Infect Dis* **27**, 2544 (2021)
